# Draft Genome Sequence of *Escherichia coli* MS499, Isolated from the Infected Uterus of a Postpartum Cow with Metritis

**DOI:** 10.1128/genomeA.00217-14

**Published:** 2014-07-03

**Authors:** Robert J. Goldstone, Richard Talbot, Hans-Joachim Schuberth, Olivier Sandra, I. Martin Sheldon, David G. E. Smith

**Affiliations:** a Institute of Infection, Immunity and Inflammation, College of Medical, Veterinary and Life Sciences, University of Glasgow, Glasgow, United Kingdom; b The Roslin Institute, University of Edinburgh, Easter Bush, Midlothian, Scotland, United Kingdom; c Immunology Unit, University of Veterinary Medicine, Hannover, Germany; d INRA, UMR1198 Biologie du Développement et Reproduction, Jouy-en-Josas, France; e ENVA, Maisons Alfort, France; f Institute of Life Science, School of Medicine, Swansea University, Singleton Park, Swansea, United Kingdom; g Moredun Research Institute, Pentlands Science Park, Bush Loan, Edinburgh, Midlothian, United Kingdom

## Abstract

Specific *Escherichia coli* strains associated with bovine postpartum uterine infection have recently been described. Many recognized virulence factors are absent in these strains; therefore, to define a prototypic strain, we report here the genome sequence of *E. coli* isolate MS499 from a cow with the postpartum disease metritis.

## GENOME ANNOUNCEMENT

*Escherichia coli* is a diverse species, and several of its pathotypes are well-characterized, including extraintestinal pathogenic *E. coli* (ExPEC). ExPEC infections of the udder and uterus of cattle have been of increasing significance, and *E. coli* is a cause of postpartum uterine disease (metritis) in cattle (1–3) and was recently proposed as a novel pathotype termed intrauterine *E. coli* or endometrial pathogenic *E. coli* (EnPEC) (2, 4). To date, genomic characterization of *E. coli* has focused on phylogenetic grouping, genotyping, and pathotyping based on the detection of defined virulence-associated factors. Among metritis isolates, phylogroup B1 strains have predominated (>65%), and these lack many virulence-associated factors typical of other *E. coli* pathotypes. Thus, we undertook genome sequencing of an EnPEC strain to define a prototype for this pathotype.

*E. coli* MS499 isolated from the uterus of an animal with metritis was previously confirmed as phylogroup B1 and possessed only *fyuA* of 17 fitness- and virulence-associated genes (4). The isolate was cultured on LB agar plates, and genomic DNA was prepared using the DNeasy blood and tissue kit (Qiagen) according to the manufacturer’s instructions. Sequencing was carried out using 454 GS-FLX and Illumina GA IIx 120-bp paired-end sequencing. The reads were assembled using Velvet (5) and the Celera assembler with the best overlap graph (CABOG) (6), and gaps were closed using unmapped 454 and Illumina reads. The assembled 447 contigs totaled 4,883,598 bp, with a G+C content of 50.7%. The assembled contigs were subjected to preliminary annotation using the RAST resource (release 70) (7) and subsequently using the NCBI annotation tool. The genome was predicted to encode 4,708 proteins. Several contigs assembled into a large plasmid showing high similarity to plasmids that confer functions in host colonization, persistence, and pathogenicity in *Salmonella enterica* serovar Kentucky and avian pathogenic *E. coli* (APEC) (8).

Frequently, *E. coli* strains of phylogroup B1 are considered nonpathogenic or poorly pathogenic (9) though strain MS499 contains many pathogenicity-associated determinants, including multiple fimbriae, iron acquisition systems, and autotransporter proteins (type 5 secretion system [T5SS]), as well as flagella. Putative toxins were absent, with the exception of an ortholog of the *hylF* hemolysin. Like many EnPEC isolates, *E. coli* MS499 is resistant to β-lactams (4), and a gene encoding a TEM-1 family β-lactamase was present.

*E. coli* MS499 from bovine metritis has many of the characteristics typifying ExPEC, i.e., elements that may contribute to pathogenicity, fitness, and resistance, and this initial genome sequence supports the previous proposal of EnPEC as a novel *E. coli* pathotype. This first reported genome sequence for EnPEC provides a resource for further defining this novel *E. coli* pathotype and forms the basis for systematic studies. Comparative genomic analyses are under way and will be reported separately in order to further define this *E. coli* pathotype.

### Nucleotide sequence accession numbers.

This whole-genome shotgun project has been deposited at DDBJ/EMBL/GenBank under the accession no. JDRV00000000. The version described in this paper is version JDRV01000000.
